# Psychological Quarterly Retrospect

**Published:** 1860-10-01

**Authors:** 


### *Psychological Quarterly Retrospect.*

The columns of the Daily Press form, as it were, a current clinical record for the psychological physician. It is well that it should be so, for the facts recorded there are common property, and the lessons to be learned from them will, it is to be presumed, be the more readily acquired, the more patent the circumstances upon which they are founded. Thus, for example, of Neglected or Unrecognised Brain Disease and Nascent Lunacy — subjects to which, notwithstanding their grave importance, but few have as yet given attention; subjects, the social aspects of which are of more immediate moment than the medical —it is from the proceedings of our courts of justice, as reported in our newspapers, that the most striking illustrations are derived. In proof of this, when such proof is needed, and in confirmation of the increasing magnitude of the subjects, we cite the following cases selected from the journals of the last three months.

Murder.—Winchester Crown Court.—(*Before Mr. Justice Keating.*)—William Henry Whitworth was indicted for the wilful murder of Martha, his wife, and his six children, at the Isle of Wight.

Mr. W. M. Cooke was instructed for the prosecution; and Mr. H. T. Cole for the prisoner.

It having been stated that the prisoner was insane, and not in a fit state to plead, the jury were sworn to determine whether the prisoner was in a fit state to plead.

Dr. Lyford, the surgeon of the gaol, was called, and he stated that he had seen the prisoner upon his first coming into the gaol, and had been in daily attendance upon him ever since, and he was decidedly not in a fit state to plead. His mind was full of delusions; his mental faculties were extinguished, as he supposed, from a softening of the brain, which must have been going on for a considerable time. He thought it would be permanent.

The Judge asked the jury to give their opinion.

The jury said they were of opinion that the prisoner was not in a fit state of mind to plead.

The prisoner was directed to be detained during Her Majesty's pleasure.—(*Times*, July 18).

In this case, the prisoner, a sergeant of artillery, had resided with his wife and family in one of the small forts in the Isle of Wight. With the destruction of his family all evidence disappeared by which the precise mental condition of the unrecognised maniac, immediately prior to the perpetration of the murders, could be ascertained; but from the evidence given on the preliminary examination before the magistrates, it seemed tolerably certain that he had shown marked symptoms of brain-disease for some time prior to the horrid tragedy. He himself was the first to announce the commission of the murders, and at the time of announcement he was labouring under manifest delusion.

Murder.—York, July 18.—Crown Court.—(*Before Mr. Baron Wilde.*) —Thomas Kirkwood, aged thirty, was indicted for the wilful murder of Elizabeth Ann Parker, at Hull, on the 23rd of April last.

Mr. P. Thomson and Mr. Lewers appeared for the prosecution; and Mr. Seymour and Mr. Waddy for the defence.

It appeared from the opening statement of the learned counsel for the prosecution, and from the evidence of the witnesses called by him, that the prisoner, who is a soldier belonging to the 29th Regiment, was in April last on furlough at Hull, and went first to stay with his sister, a Mrs. Todd, at Hull, and afterwards lodged with a person named Taylor. While staying with his sister he made the acquaintance of the deceased, Mrs. Elizabeth Ann Parker, a young widow, who lived with her mother, Mrs. Sleight, and stayed at the house of Mrs. Sleight. A rumour having reached them that he had overstayed his leave of absence, they got rid of him, and he then went to lodge with Taylor. He continued to call at Mrs. Sleight's, and on the morning of the 23rd of April he went there as early as eight o'clock, and remained there till about eleven, talking with Mrs. Parker. At eleven he went out to a neighbour's named Burnham, and asked for a pipe, and then went back to Mrs. Sleight's. There he bade good-bye to Mrs. Parker and gave her a kiss, and suddenly put his arm round her neck and cut her throat with a razor. Mrs. Burnham heard her name called twice, and then a cry of murder, and on looking out of her door saw the prisoner and Mrs. Parker scuffling together, she having her hand on his shoulder, and he attempting to get away. Mrs. Parker was covered with blood, and she saw the prisoner make his escape from her and run away. On going to Mrs. Parker she was found with a deep wound at the side of her neck, which bled profusely, two large veins having been divided. At the time a surgeon named M'Mellan was passing, and he, with the assistance of another medical gentleman named Carnley, succeeded in tying the veins and stopping the bleeding, but the deceased was so exhausted that she sank and died about four o'clock the same day. Near where the scuffle had taken place the blade and haft of a razor were found, broken and saturated with blood, and this razor the prisoner had borrowed from Taylor to shave with the night before. Before she died Mrs. Parker made a declaration that the prisoner called at eight o'clock that morning, and sat talking for three hours. She had no quarrel with him. He said nothing before he attacked her. He was not courting her, and she knew no motive why he should injure her. He had given her a kiss once or twice before, when he bade her good-bye. It appeared from Mrs. Sleight's evidence that the prisoner was in the habit of bidding good-bye, and saying he was going away next day; and she corroborated the statement that her daughter had not countenanced any attentions from the prisoner. After leaving Mrs. Sleight's house it appeared the prisoner went to his lodgings at Taylor's, with his handkerchief wrapped round his hand, which was bloody, and he gave as an excuse that he had been drilling, and had got his hand hurt with a bayonet. He then washed his hands, and went out. He seemed very excited, and the worse for liquor. He was taken into custody a little before twelve o'clock, sitting by the roadside with his head on his hand and his clothes bloody. The witnesses all agreed that he had had nothing to drink before he attacked the deceased. There was no evidence to show that he had had any liquor afterwards, and it appeared that he was habitually a sober man.

For the defence, witnesses were called who proved that he had a sister, who was insane and who died so; that he had a brother who was not considered to be sound in his mind, and that the prisoner himself, when eleven years old, had his head shaved, and used to go about in a nightcap, and his manner and demeanour then got him the nickname of "Fond Tom." When he grew up he was moody, and used to sit alone with his head on his hand for hours; he was always depressed, and in low spirits, and dull. After he entered the army he went to India, and there had the country fever and a sunstroke, and two of his comrades, who served in the same country, swore that he was unlike other men; he never joined in the sports of the men, and used to keep aloof. He was eccentric and peculiar, got up at nights and walked about muttering to himself, and was then considered to be not quite right in his head. When he came home again his sister swore that he used to jump out of bed at nights, and walk about the house muttering something to himself which she could not understand. Sometimes he would burst out laughing, and when asked what he was laughing at, he would say, "Nothing." She got so alarmed about him that she got the errand boy employed at the house to sleep with him, and this boy gave similar evidence as to his jumping out of bed and walking about at night muttering. Sometimes he would sit at the bedside making faces when no one was there. His sister got so alarmed that she wrote to his captain to recall him, and got rid of him out of her house.

The motive suggested by the prosecution was, that the prisoner had said he had met the deceased the night before with a man with moustaches, and that he was influenced by jealousy, but there was no proof whatever that the deceased had been out with any man the night before, and her mother was confident she had not, and this was urged for the defence to be a mere insane delusion.

On the grounds that insanity was hereditary in his family, that he had early shown symptoms of this terrible malady, and that his conduct up to the period of this occurrence had been such as to make those who associated with him believe him not to be sane, and that his attack on the deceased appeared to be quite without motive and when he was proved to be sober, it was contended that the murder was committed by a man who was insane, and not legally responsible for his acts.

The learned Judge, in a most clear and able manner, summed up the evidence. He directed the jury that anything like eccentricity or peculiarity of manner was not sufficient to excuse a man from the penalties of the crime of murder. It might well be that such a man could well understand the distinction between right and wrong. But they must consider whether the man's mind was proved to be so unsound that he did not know the character and quality of the acts that he was committing. If they thought so they must acquit him on that ground; if not they must find him guilty.

The jury retired, and, after an absence of nearly an hour, returned with a verdict of *Not Guilty*, on the ground of insanity.

The prisoner was then ordered to be confined during Her Majesty's pleasure.

During the trial the prisoner, who appeared in his uniform, stood with his head bent down and leant on his hands. He had a great deal of very thickly-set hair, which appeared almost like a wig about his face. Until the sentence was pronounced he never looked up.

Suicide.—An inquest was held yesterday (Aug. 6) at the Ship Inn, Redcliff-hill, Bristol, before Mr. J. B. Grindon, city coroner, on the body of Mr. George Haynes Hinchcliffe, one of the coroners for the county of Stafford. The circumstances attending the case were of a melancholy and almost romantic character. The deceased gentleman was married only as lately as Wednesday last at West Bromwich to a lady of equal position in society, to whom he had been for some time previously engaged, and in course of that day the newly-wedded pair arrived at the Queen's Hotel, Clifton, on their trip for the honeymoon. Nothing strange was observed in the manner of the bridegroom until he went to his wife's chamber some time after she had retired for the night, and shortly afterwards reappeared and requested to be provided with another bedroom. The house being full, Mr. Hinchcliffe was told that he could not be accommodated, and he then left the Queen's and proceeded to the Sedan Chair Tavern, on the Broad-quay, where he slept for the night. His wife, alarmed at his strange behaviour, telegraphed for her brother, Mr. Fereday, of West Bromwich, who arrived in the course of the following day, and Mr. Hinchcliffe was sought out and prevailed on to return to his wife. He dined with her and her brother the same evening, but again left the Queen's, and there is reason to believe that he wandered about all night. On Saturday he took lodgings for the night at the house of Mr. Price, grocer, of Thomas-street, and there committed suicide under the strange circumstances disclosed in the subjoined evidence.

William Bateman Reed deposed,—I am the proprietor of the Queen's Hotel, Tyndall's-park. On Wednesday evening last the deceased arrived at my hotel with a lady; they had the appearance of a newly-married couple. The body I have seen at the General Hospital is the body of the gentleman. Late in the evening the chambermaid informed me that the bridegroom retired to his room some time after the lady, and that he came out shortly afterwards and asked for another bed. We could not accommodate him, and he left the house without any luggage. The next morning the lady telegraphed to her friends, requesting them to come down. The gentleman returned on the following morning, and asked to see the lady, but she refused to do so unless in the presence of her brother, Mr. Fereday, to whom she had telegraphed, requesting him to come down, as she was in extreme danger. I removed the lady to my private house, as she stated that she feared her husband had gone insane. In the afternoon a man in the garb of a sailor came for Mr. Hinchcliffe's luggage, but I did not then let him have it. I went with the brother-in-law and found deceased at the Steam Packet Tavern. He returned to our house, and dined with his wife and brother-in-law. He appeared to be very low in spirits. The brother-in-law told me he could not discover any reason why they should separate. I spoke to the deceased between 10 and 11 o'clock, and he told me that he had gone into his wife's bedroom on the Wednesday evening and told her that they could not be happy together. She said, "Good God! you had better leave the room," he did so. This, he said, was all that passed. On Thursday evening he left our house, and went, as I ascertained, to the Sedan Chair, and slept there that night. He called again at our house on Friday and Saturday, on which latter day his wife and brother-in-law had left.

Walter Thomas, grocer, of 99, Thomas-street, Bristol.—I saw deceased on Saturday night a little before 12 o'clock. He asked me if I had a spare bed. I told him I did not let lodgings, but he could get a bed at the Queen's Head, close by. He said he did not want to go to an inn, and, after consulting my wife, I agreed to accommodate him with a bed. I accompanied him to his bedroom. He looked to see if there was any water in the bottle, and I got him some and he drank it. He made no particular observation; he said he would breakfast with us the following morning. He asked if I could let him have a razor and brush in the morning, and I said I would. I went to bed about a quarter after 12, and was roused the following morning, about 2 o'clock, by the police.

Police-sergeant Foot.—About 20 minutes to 2 on Sunday morning I saw a man at the top of Mr. Price's house, who said he had been robbed, and on my ringing the bell he told me it was all right, as he had found what he had lost. Another officer came up, and we asked him (deceased) his name and residence, but he refused to give them, and said he did not believe we were policemen at all. Several persons gathered round, and I said I would call up Mr. Price and see who his lodger was. We had had information of a man having escaped from Dr. Fox's Asylum. I went away and returned in a few minutes, when I saw the man holding on by the window-sash. I cautioned him of his danger, and in a minute or so his hold gave way, and he fell into the street. I immediately picked him up, and found him apparently dead. In two or three minutes after rousing Mr. Price, and getting a shutter to put the body on, we took deceased to the General Hospital.

Police-constable 185 confirmed the evidence of the last witness.

Mr. Dowling, house-surgeon at the Bristol General Hospital.—I was at the hospital when deceased was brought there on Sunday morning. He was then dead. His skull was fractured in such a way as to cause his death. He had a fracture of the left thigh also. I have made an external examination of the body. Deceased was suffering from hernia. It was not in a dangerous state. He wore a truss. There are many popular prejudices that hernia is more serious than it really is, and, if viewed in that particular light, a sensitive man's mind might be affected by this disease.

The Rev. George Frederick Wade, incumbent of Eastoft, Yorkshire, brother-in-law of the deceased.—I never knew deceased anything but perfectly sane. I married him a few days ago at West Bromwich. He left for Clifton. I am certain he is the person now lying dead at the General Hospital. I have known deceased for nearly 20 years. He was considered by all who knew him as the ideal of all that was honourable, truthful, and gentlemanlike. He succeeded his father as one of the coroners for Staffordshire, having been elected by an overwhelming majority. He was exceedingly nervous. I have known him faint at matters which another man would only laugh at. I saw his brother-in-law, Mr. Fereday, on Saturday, who told me of all the strange things that had happened up to that time. I was astonished, as when I saw him and his wife leave I thought what a happy couple they were. Deceased was very excitable, particularly sensitive of anything like blame. The rupture which has been spoken of took place while he was hunting. There was nothing to have prevented him and his wife from living happily together. His wife was an exceedingly nervous lady, and no doubt that has made matters worse. I do not believe the deceased would have destroyed himself had he been in possession of his faculties. He was quite alive to the sin of self-destruction. I respected him as I respect few persons in this world, and loved him dearly. He was 33 years of age.

Frederick Kalkroven.—I keep the Sedan Chair, Broad-quay. On Thursday evening deceased came to my house in a cab, and ordered a bed. He took no refreshment of any kind. He was very quiet. He went to bed a little before 12. He brought a large carpet-bag and two coats with him. Nothing about his manners betrayed insanity. He went out in the morning without taking any breakfast. He did not return that day. He left his bag open in the bedroom. Between 5 and 6 on Saturday evening he came with an officer and said he wished to pay his bill. He was very quiet at the time. He paid his bill, and left, as I understood, for the railway station.

Mary Ann Tovey, of Waterloo-court, Thomas-street.—I saw deceased on the housetop of Mr. Price on Saturday night for some time. He said he wanted a policeman. I told him there were two there. He said I was trying to "gammon" him; they were not policemen. He talked like a man deranged. I saw him fall from the window into the street.

Police-constable Buller.—On Saturday afternoon deceased came to the Central-station and said he had lost his carpet-bag at a public-house on the Quay. I went with him, and found his bag at the Sedan Chair. He then requested me to get a cab for him, as he said he was going by the 6.45 train to Birmingham. I did so. He gave me his name as Mr. Hinchcliffe, of West Bromwich. He talked in a very strange way at times to me, and I thought he was deranged.

The Coroner having summed up, the jury without hesitation found that the deceased destroyed himself while labouring under temporary insanity.—(*Times*, Aug. 17.)

These are marked examples of a class of cases which, from being neglected or unrecognised, play immense mischief in society. If we could flatter ourselves that, sooner or later, such cases, after the fashion of those we have just related, invariably ended in some criminal act which brought them within the grasp of the law, or which terminated the case summarily, we might suppose that the evil, in one, and an exceedingly contracted and somewhat inhumane sense, worked its own remedy. But that is far from being the fact. Crime and suicide are but two of the many unhappy results to which neglected or unrecognised brain-disease leads. Most frequently, within the sacred circle of domestic life, the undetected mischief gives rise to untold-of misery and ruin, until it terminates, perhaps after long months of wretchedness, in unmistakable lunacy.

If we would lift the veil still higher from this painful but most important subject, we need but revert again to the criminal records of the quarter. On the 23rd of July, Thomas Hopley, a schoolmaster, described as a man of high attainments, was tried, at the Lewes Assizes, for the manslaughter of Reginald Channell Cancellor, a boy of fifteen years of age, and one of his pupils. Hopley received for the charge of the boy a stipend of 180*l.* per year. The lad exhibited no aptitude for learning, and was looked upon by his master as being unusually obstinate; and in April he had written to Cancellor's father, telling him that he had tried every means in his power to conquer the boy's obstinacy, but without avail, and that the only thing less to be done was to resort to strong measures of corporal punishment. The father gave permission for Hopley to act as he thought fit. This permission Hopley construed in the very fullest sense, and adopted a course of treatment towards the boy, almost unparalleled in its brutality, and which ended in the lad's death. It was proved on the trial that, for a period of nearly two hours, the prisoner beat Cancellor with a skipping-rope and a stick, and that the boy either died under the punishment or very quickly after it.

The following statement was made by the prisoner before the magistrates on his preliminary examination, and was read at the trial:—

"The deceased was a very peculiar boy, and was not only very obstinate, but was also actuated by a determination not to learn anything. Although between fifteen and sixteen years old, he did not know the difference, or pretended not to know it, between a shilling and a sixpence or a fourpenny piece. He communicated with his father, and he considered that he had his sanction for what he did, and, feeling that it was absolutely necessary that he should master the boy's propensities, he resolved, with great regret, to do so by severe punishment. He was in one of those fits of obstinacy on the day in question, and he admitted that he beat him until he subdued him, and he said his lesson rapidly and correctly. After this the prisoner said the fit again returned, and the deceased refused to go upstairs or to undress himself, and feeling that if he had given in it might have the effect of ruining the boy for ever, he again punished him, and succeeded in subduing him, and the deceased expressed himself grateful and went to bed. The prisoner admitted that he used the rope and the stick, but said he only beat the deceased about the legs and shoulders, and he had no other instrument to make use of, he being so averse to corporal punishment that he had not even so much as a cane in the house. The statement concluded by an assertion by the prisoner that he was not at all in a passion or in anger when he inflicted the punishment upon the deceased, but that he felt he was doing his duty, and that he repeatedly requested the deceased to give in, and spare him the pain of inflicting further punishment upon him.

The body of the unfortunate lad was examined, six days after death, by Mr. Prescott Hewett, who deposed as follows:—

He made a post mortem examination of the deceased on the 28th of April, assisted by Dr. Willis and Dr. Holmes. When he first saw the body it was completely covered, so that no part but the face was visible. There were white kid gloves on the hands, and the legs and feet were covered with apparently men's stockings, which reached half way up the thighs. Upon removing the coverings and examining the body, he discovered that the legs and arms were of a dark livid colour, and swollen from extravasated blood. He cut through the skin, and then ascertained that there was a very large quantity of blood extravasated into the cellular membranes underneath. Under the skin of the palm of one of the hands there was extravasated blood three-quarters of an inch in thickness, and the cellular membranes under the skin of the thighs were reduced to a perfect jelly—in fact, all torn to pieces and lacerated by the blows that had been inflicted. The injuries must have been inflicted by some heavy blunt weapon, and the stick that had been produced was an instrument calculated to have inflicted such injuries. The rope, in his opinion, was calculated to make the bruises, and the stick to have produced the lacerations to which he had referred. On the right leg of the deceased he observed two wounds about the size of a sixpence, and an inch in depth, and he was of opinion that these wounds might have been occasioned by a job or thrust with the pointed end of the stick that had been produced. The head of the deceased was large, and exhibited the appearance of his having suffered from water on the brain, and this turned out to be the case when the head was opened.

The Lord Chief Justice.—Would this condition of the brain account for the deceased being of defective intelligence?

Witness.—It certainly would do so.

Dr. Hewett, in conclusion, said that from the appearances he observed, he was satisfied that considerable violence had been used to the deceased, and he came to the conclusion that his death was caused by a shock to the nervous system, and by the large quantity of blood extravasated in the cellular membranes. He also expressed his opinion, that from the evidence before the Court, and the statement made by the prisoner, that the body of the deceased was stiff when he first saw it in the morning, that the death took place about twelve o'clock on the previous night.

A verdict of *Guilty* was returned, and Hopley was sentenced to four years' penal servitude.

Here, then, we have an example of a man of reputed high attainments, a schoolmaster of mark, regarding the incapacity of a semiidiot as sheer obstinacy! But, even supposing he had been right, how shall we characterize the horrible brutality of the method adopted to overcome the lad's perversity? It is almost inconceivable that at a time (to take no higher ground) when Rarey was showing to crowded audiences in London the worse than futility of endeavouring to break in animals by means of physical punishment, that an educated man, within a few miles of the spot where the horse-tamer was lecturing, should have thought that by means of the rod he could conquer and train a supposed obstinate boy! Rightly did the *Times* say of Hopley:—

"There is nothing to be said for this man. Every one must feel that four years of penal servitude is by no means too severe a sentence for the crime he has committed. It is true that the boy never ought to have been put under his management, that his was an exceptional case, and ought to have been medically treated. It may be true that Hopley thinks that severity is the only way to break in stubborn boys. But to beat a boy for two hours with a thick stick and a skipping rope, to macerate him, to 'prod' him, in private and at midnight, is not discipline, but murder. All private punishment should be severely discountenanced, but it would be absurd to speak of this as punishment; it was a deadly attack, followed by a naturally fatal consequence. This case has come out much worse than the preliminary investigation prepared us to expect, and we hold it up as a warning, not less to parents than to schoolmasters."

To return, however, to the question with which we started:—The cases we have quoted teach us the extraordinary ignorance which is prevalent among all classes of society concerning the significance of some marked forms of mental disease. Had a degree of perversion corresponding to that witnessed in the functions of the brain been manifested, in the different cases, in the functions of the heart, the intestines, or the muscular system, the need of the physician would have been at once felt and his aid sought. It is not until the public learn to look upon the signs of disordered brain-function in the same light as those of any other function, that we shall miss cases such as those we have just recited among the records of our courts of justice, or mitigate the less apparent evils occasioned by neglected or unrecognised brain-disease. Lelut has well said that "madness is not a thing apart; all madmen are not under the protection of the asylums which are devoted to them. From complete or philosophical reason to delirium truly maniacal there are innumerable degrees, of which it would be advantageous that every man should have a general knowledge, lest anger or vengeance should usurp the place of an indulgent pity—a pity which each one may at one time or other have required, and which he may require again."

While, however, we insist upon the importance of recognising Neglected and Unrecognised Brain Disease, as a source of certain social evils, and one deserving of more accurate study than it has yet received, we must not err by exaggerating that importance at the expense of other and equally weighty considerations. If the current events of the quarter have furnished several striking illustrations of this subject, neither have they been wanting in other matter of hardly less interest to the practical psychologist. Thus, *suicide* forces itself upon the attention. The case we have already cited was the result of disease, and demands our pity; but many cases have been recorded during the past quarter, which, from the comparative triviality of the causes inducing the act, indicate the existence of a most unhealthy tone of thought upon the subject among certain of the metropolitan classes, which cannot be too greatly reprobated. It is not too much to say, that in England suicide as a rule is divested of any sentimentality, and wears a very commonplace and unattractive aspect. It is with our population looked upon truly as a last resort, and not as a legitimate mode of escape from the first serious cross which may happen to occur to an individual. Suicide is, however, just one of those subjects which are most apt to receive a gloss from unhealthy sentiment, and so impose upon immature and too mobile minds. It, therefore, behoves the public on the first signs of a reaction in favour of suicide among any class of the population, to crush it at once by utter and immediate condemnation. In a question of this kind, we feel assured that public opinion would be all-powerful.

No organ of the press does such good service in this matter as the chief of the cheap journals—the *Daily Telegraph*. The mode in which this journal hunts down suicide deserves the warmest commendation. Admirable in tone, sound in object, and brilliant in quality, the articles which it has devoted to the subject in the past quarter were fully calculated to effect that good, which from their nature, and the enormous circulation of the journal, might be hoped for. We shall reproduce these articles, as well for the lessons they contain as for the interesting illustrations they afford of the feelings of the most important organ of the cheap daily press upon a most painful and disheartening social evil.

It is probable that, were the statistics of suicide analyzed, and were we placed in a position to fix with certainty on the circumstances which have led to every attempt at self-murder, we should find that a large proportion, if not a majority, of these criminal acts were due to the most trifling causes. Your deeply-dyed criminal seldom tries to hang himself to the bars of his cell-window, or to dash out his brains against its stone walls. When, indeed, he does attempt *felo de se*, it is not because he is told that he is about to be transported for life, but because the governor has stopped his ration of boiled beef, or the turnkey has reprimanded him for not folding his counterpane properly. A woman who has cut the throats of half-a-dozen children is committed for trial, and goes away quietly enough in the van to Newgate, whereas a poor "drunk and disorderly," who has been remanded to the police-cell through inability to pay a five-shilling fine, straightway proceeds to hang herself in her garters, after the manner of the unfortunate Miss Bailey. A shop lad accused by his master of embezzling a few halfpence will often cast himself into the canal, whereas the rogue who has forged for thousands of pounds, and beggared dozens of families, receives his sentence of penal servitude without murmuring, and takes to chairmaking or matweaving, under the auspices of the prison taskmaster, quite blithely. Many a servant-girl, whose mistress has reproached her with a propensity for ringlets or crinoline, or who has had a "few words" with her "young man" in the Coldstreams relative to her flirtation with some dapper constable in the P division, rushes to the chemist's shop, purchases some oxalic acid, and crams it down her throat. In moral and religious tracts suicide is generally the orthodox termination to a career of female frailty. "Drowned! drowned!" is the fifth act to the drama which commences in a villa at St. John's-wood; but any intelligent police-constable or divisional surgeon will tell us that the suicidal element enters but in an extraordinarily minute degree into the phases of the social evil. Now and then some wretched creature will fling her bonnet and shawl on the pavement, and jump over the parapet, or rush down the steps of a bridge into the water; but in nine cases out of ten it will be found that the predisposing causes of the rash act have not been misery or want, or remorse at a life of sin, but that the unhappy creature has been drinking rather freely, or has had some paltry squabble with a sister Cyprian or her landlady. Of course, we leave on one side the self-slaughters that are perpetrated through suicidal mania, through constitutional hypochondria, through deliberate design—as when a man sees no way out of his difficulties, and calculates, as has often happened, that he may better his position by killing himself. But there have been suicides for the toothache. There have been suicides because it rained—because a new bonnet had not come home from the milliner's. There have been suicides through emulation—suicidal epidemics which form, perhaps, one of the most curiously obscure diseases of the mind of which a mental pathologist could treat.

Self-destruction, unhappily, has at all times been very prevalent among the young, more especially among the weaker sex; and the causes are almost invariably the same—crosses in love and quarrels with parents. The latter reason is so frequent that it developes itself even in children, and boys and girls of ten and twelve have been known to commit suicide to revenge themselves for a scolding, or to avoid a punishment with which they have been threatened. Nine-tenths of the painful domestic tragedies which plunge respectable families into irremediable despair are attributable to one fatal cause—temper. There is temper, often, on the part of the parent who too harshly censures or corrects a child. There is equally temper on the part of the son or daughter who rebels against a fancied usurpation or overstraining of parental or maternal authority, who is unable to struggle against the superior power of the head of the family, and who—determined that revenge shall not be quite impotent—invokes the aid of the water-butt or the laudanum-bottle. Not many days since, our columns contained a report of a most lamentable event that occurred in the family of a respectable tradesman at Plymouth. The father had some wretched squabble with his daughter, a girl of nineteen, about the price of a pair of boots she had sold. His temper became ungovernable. He took a rope and beat the girl severely. She, stung to frenzy by the humiliating chastisement, rushed up stairs, flung herself out of a window, and dashed her skull to atoms on the pavement. We daresay that many who read the report of the inquest were moved to feelings of the strongest indignation against a man who seemed heartless and brutal enough to flog a girl of nineteen with a cord; but, when the sad history came to be analyzed, it was found that the girl had before threatened to commit suicide, that this last conflict was only the end of a series of quarrels with her father, and that she was obdurate and rebellious. There were faults on both sides, and on each the error was temper.

Under the head of "romantic suicide and attempt at suicide," our readers have quite recently perused another sad story, which bears with equal force on our remarks. Mr. Brent, the coroner, has been holding an inquest on the body of a young girl only seventeen years of age, who, in company with a female friend about the same age, threw herself into the New River, at Highbury Vale, on Thursday last. The two girls were children of most respectable parents, and having formed an acquaintance at a Sunday school treat two or three years since, had become most intimate friends. They wrote love-letters to each other —as girls will do before they have something better to love—exchanging small presents and locks of hair. The deceased girl appears to have been very fond of "pleasure"—that is, of balls and junkettings. On the 25th of June she remained from home all night. In great alarm, her father called on the family of her young companion, thinking they were together. Only one, however, and that one not his daughter, was at home. The deceased, however, came home, and her mother is said to have struck her for attempting to speak to her friend. What other scenes of bickerings and harshness may have preceded or followed this act, it is out of our power to determine. Finally, the two young women ran away, with—God help them!—sixpence in their pockets. The deceased had told her mother it was the last she would ever see of her. They walked some time and got very hungry, when the deceased proposed to buy arsenic for both. They walked beyond Edmonton and back into London. They lived on one shilling and threepence, which a gentleman gave them in charity, until Thursday morning—they had run away on the Tuesday. The girl who survives, asked her friend, if she would return home. She replied that she never would. They walked to Highbury Vale, and joining hands, and shrieking, "Oh, love! love!" jumped into the New River. Was there ever a tale "so sad, so tender, and so true?" The girl who lives was rescued by a gentleman who heard the cries of the unhappy pair. A brave plasterer—all honour to him—who was at work in a neighbouring building saw a shawl floating in the water, jumped in, and brought—alas: nothing but a dripping corpse to land. Every means of resuscitation was tried, but in vain. The girl was proved to have been good and virtuous beyond suspicion. Nothing but this unhappy temper had conspired to render her home miserable, her parents bereaved, herself a guilty suicide. We trust that the result of this most tragical event will be, for the remainder of her life, an awful but salutary warning to the young woman who has been providentially delivered from a watery grave; but should it not also be a warning as pregnant with example to parents who treat their children with undue harshness, and, in their correction of the imprudences of youth, have recourse not to reason and kindness, but to revilings and blows?—(*Daily Telegraph*, July 6.)

It can scarcely have escaped public notice that for a considerable time past, and especially during the month now ending, the London magistrates have been constantly engaged in dealing with cases of suicide. It may or may not be a question whether some mystery of nature exists in connexion with a mania of this kind, whether atmospherical or astrological influences are at work, or whether philosophy may find a cause for so deadly an autumnal epidemic. One of the modern magi affirms that individuals born under the sign of Saturn, when the sun is in Hyleg, are liable to attempt self-destruction; but so far as we have investigated the promptings of metropolitan make-believes at *felo de se*, we discover them to be, firstly, drunkenness; secondly, imposture; and thirdly, despair—this last being in all cases accompanied by a strong development of mental disease. It is often difficult, however, to draw the line at which, in consequence of this morbid affliction, personal liberty should be restrained. The literary gentleman who deprived himself of life a few months since, did so in the very midst of his ordinary intellectual tasks, and stopped reviewing a book in order to cut his throat. There can be no doubt that the mind of the unfortunate west-country coroner who threw himself out of window was mortally affected. The poor clergyman whose death we on Wednesday recorded, was, of course, at least temporarily insane. Concerning this class of suicides we are not proposing to treat. They belong to the psychologists, the physicians, and the humane guardians of lunacy. But there are others which have been painfully and repulsively obtrusive of late. The sottish artisan, who has squandered his wages in bestial excess, and has reduced himself to a state of inhuman bewilderment, staggers to the water's edge and drops in, perfectly aware that the policeman, whose bull's-eye is upon him, will come to the rescue and lodge him safely in the station-house. The draggle-tailed hussy who has pawned her husband's clothes, beaten her children, received two black eyes in a fight, and saturated herself to the blood and to the brain with gin, howls down the steps of some bridge, sure of attracting attention, and walks into the river like a timid bather, just far enough not to be out of reach. She is certain to shriek before she takes her drenching, and it may be noted, as a fact for Benthamites, that these intoxicated shams of suicide never take place except within sight or call of a crowded thoroughfare. The genuine outcast, the desolate creature who wanders to the Bridge of Sighs, the hopeless criminal resolved upon anticipating justice, seeks the loneliest niche on the loneliest bridge, or the midnight obscurity of Hampstead Heath, or a private room in a retired hotel; the brawler, savage with himself, and the harridan, who has nothing left to her but a mimicry of self-drowning, or strangulation with her garter, habitually yells loudly enough before enacting the hideous farce, whether in the shallows of the Thames or in the cell of a police-station.

We do not assume that any magisterial efforts will ever give meaning to the menace of Sir Peter Laurie, and "put down suicide." Nor would we recommend, as one way of aiming at this result, the course adopted formerly by the municipal Government of New Orleans, in which city, for three consecutive months, the corpse of every self-murderer was hung up naked and publicly scourged—an indignity which speedily toned down the romantic aspirations of young gentlemen and ladies ambitious of a poetical notoriety after death. Captain Langley records that in Sindh, when a person attempted to drown himself, he was taken out of the water, kicked seven times, and set free; but it might be dangerous to arm the night police of London with so anomalous a prerogative. Yet is there no possible method of checking this horrible and disgusting mania? It has been demonstrated that the ancient and barbarous custom of cross-road graves and unconsecrated burials is wholly inoperative. Verdicts of "Temporary insanity" almost invariably, except in the cases of suicides who have slain others before slaying themselves, relieve the dead from this unchristian insult. But we are speaking now of impostors who intend only to run the risk of a soaking, or tighten a handkerchief round their necks, in order to rouse sympathy or to gratify some instinct of drunkenness. Too often the magistrates, in their benevolence, allow them to go unpunished, after an admonition wasted upon callous ears. Now, where it is established that the attempt was a sham, the severest punishment ought to be inflicted, as upon rogues and vagabonds of the worst description. Under other circumstances, when poor girls who have been seduced, or semptresses out of work, or children dreading chastisement, have essayed, more or less resolutely, the destruction of their own lives, they ought never to be set free without undergoing more or less of penitentiary discipline. We do not mean to imply that they should be classed with thieves, disturbers of the peace, or criminals of any kind; but they should indubitably be confined for a longer or shorter period, kept to moderate labour upon the plainest wholesome food, and receive a course of admonition such as might drive out of their minds the idea of suicide. A repetition of the offence must invariably be considered a serious infringement of law, and visited with the penalties either of a House of Correction or of a lunatic asylum, the latter being, perhaps, the more effectual. Moreover, the public, we fear, is often too ready with its subscriptions in cases where young women, having led a dissipated life, and dreading the struggles of poverty, seek for sympathy by exhibiting themselves in a police court, trembling from the effects of a plunge in the river. Too much discrimination cannot be observed with regard to these appellants to the benevolence of society. The Penitentiary or the Reformatory is the fit receptacle of every one whose mind has been so completely overpowered, and whose conscience has been so fatally drugged, as to risk the death of felony rather than endure the ordinary troubles of the world. A night in a police cell, an exhortation from the magistrate, or even a week's visitations by the chaplain, can hardly suffice for the cure of a moral malady so fearful. But, as we have said, a distinction must be drawn between serious and deep anguish, provoking to the rashness of self-murder, and that maudlin self-abandonment which leads the drunkard to the river's edge, to be dragged out by a waterman or a constable. The sots and vagrants who perpetrate this offence are fit subjects for the stocks and pillory, and the law, while not pretending to deal with human nature as with a vulgar mechanism, ought to treat a pretended suicide as it would treat a fortune-telling gipsy, or a begging-letter impostor.—(*Daily Telegraph*, Aug. 31.)

In further illustration of this subject, we quote the following singular but instructive case:—

Attempted Suicide and Murder—Winchester Assizes.—Crown Court, July 18.—Robert Simpson (a private in the Rifles) was indicted for cutting and wounding Sophia Rowe, with intent to kill and murder her, at Winchester.

Mr. Gunner was counsel for the prosecution.

This was one of the most extraordinary cases ever heard. It appeared that Rowe was the daughter of a brickmaker named James Rowe, who lived at Stowmarket, Suffolk. She had come to Winchester as a servant. In December last she was out of place. She went to the Painters' Arms in Winchester to assist the landlady, a Mrs. Watlen, and she remained at that house until Saturday, the 12th of May. In the meantime she had become acquainted with the prisoner, who was 22 years of age, and they kept company together. On a Thursday and Friday night they slept together, and on the Friday morning, before they got up, the prisoner said he was tired of being a soldier, and should cut his throat, and he requested Rowe to borrow a razor for him. She told him she would not borrow one. He then became very unhappy, and the girl gave him 1*s.* 6*d.* to purchase a razor with. They got up, and in the course of the day the prisoner went out and bought a razor, and he gave it to Rowe, who locked it up in her box. They remained at the Painters' Arms all the rest of the day. When they went to bed, between ten and eleven o'clock at night, the prisoner asked Rowe to put the razor under the head of the bed, and she took it out of her box and put it under the bolster. Soon after they were in bed another lodger, named Rachel Welby, came in and got into the same bed with them, and slept with them all night. On the Saturday morning Rachel Welby got up a little before seven o'clock, and while she was dressing she heard Rowe say, "Do it, dear; do it, dear." After she had gone down, Rowe stated that the prisoner said he was determined to cut his throat; she endeavoured to dissuade him, but he said he would do it. Rowe, who was very fond of him, said if he cut his own throat he should cut hers first. He said he had not power to do it. Rowe said if he would not cut hers he should not cut his. The prisoner then asked her to lay her arm down for him to put his head on. She laid her arm down, and he placed his head upon it. He at that time had the razor in his hand, and he cut her throat, and then he cut his own. It would seem that some noise was made, and Mrs. Watlen went into the room, and saw them both lying in bed on their backs with their throats cut. Rowe said, "Good-bye," and the prisoner said, "This is love." Rowe then said, "I shall die happy." Mrs. Watlen asked Rowe who had done it, and she pointed to the prisoner. Mrs. Watlen instantly sent for Mr. Buckle, a surgeon, who found them in the manner before described by Mrs. Watlen. Their throats were bleeding very much, and the woman was just fainting from loss of blood. The girl's throat was more severely cut than the prisoner's. The surgeon immediately took the requisite steps, and saved the lives of both parties.

The learned Judge, in summing up, said that if the jury believed the evidence, they must find the prisoner guilty, because, had the woman died, he would have been guilty of murder. He supposed the people had been reading novels. It was a most shameful and wicked act. Even if the girl had asked him to take her life, he had no right to do so.

The jury found the prisoner *Guilty*.

Judgment of death recorded.

